# Effects of Elevated CO_2_ and Heat on Wheat Grain Quality

**DOI:** 10.3390/plants10051027

**Published:** 2021-05-20

**Authors:** Xizi Wang, Fulai Liu

**Affiliations:** Department of Plant and Environmental Sciences, Faculty of Science, University of Copenhagen, Højbakkegård Allé 13, DK-2630 Tåstrup, Denmark; xiwa@plen.ku.dk

**Keywords:** elevated CO_2_, grain quality, heat stress

## Abstract

Wheat is one of the most important staple foods in temperate regions and is in increasing demand in urbanizing and industrializing countries such as China. Enhancing yield potential to meet the population explosion around the world and maintaining grain quality in wheat plants under climate change are crucial for food security and human nutrition. Global warming resulting from greenhouse effect has led to more frequent occurrence of extreme climatic events. Elevated atmospheric CO_2_ concentration (eCO_2_) along with rising temperature has a huge impact on ecosystems, agriculture and human health. There are numerous studies investigating the eCO_2_ and heatwaves effects on wheat growth and productivity, and the mechanisms behind. This review outlines the state-of-the-art knowledge regarding the effects of eCO_2_ and heat stress, individually and combined, on grain yield and grain quality in wheat crop. Strategies to enhance the resilience of wheat to future warmer and CO_2_-enriched environment are discussed.

## 1. Introduction

Global atmospheric concentration of carbon dioxide (CO_2_) is expected to reach levels of 420 ppm (RCP2.6) to 1300 ppm (RCP8.5) by the end of this century (IPCC, 2013) with a concomitant rise in mean global temperature of about 2 °C by 2050 (IPCC, 2014). Extreme climatic events (ECEs), such as heat waves and droughts, which often affect plant growth and pose a growing threat to natural and agricultural ecosystems, are predicted to increase in frequency and severity in many cropping areas [1,2]. Wheat is one of the world’s major food crops with an average annual global production of over 750 million tons from 2015 to 2019 (http://faostat.fao.org/ (accessed on 14 May 2021)). It is considered an important source of starch and energy. Wheat provides significant amounts of important nutrients, including proteins and mineral elements, as well as other components that are beneficial to human health, such as vitamins (especially vitamin B), phytochemicals and dietary fibers [3]. The effect of elevated atmospheric CO_2_ concentration (eCO_2_) on wheat grain yield and grain quality has been well studied [4,5,6]. A general increase in grain yield and a reduction of grain quality of plants grown under eCO_2_, especially the decrease of nitrogen (N) concentration and thus also protein contents, have often been reported, leading to the conclusion that eCO_2_ potentially exacerbates the prevalence of “hidden hunger” for human nutrition [7,8].

Another environmental factor, heat waves, limits wheat yields globally [9]. Air temperatures have increased since the beginning of the century and global temperature is predicted to increase 1.5–5.5 °C in the next 50 to 75 years [10]. Such increases in the temperature can lead to heat stress, a severe threat to wheat production, particularly when it occurs during reproductive and grain-filling phases [11]. Previous studies showed that exposure to temperatures above the optimum temperature for wheat at anthesis and grain filling stage (12 to 20 °C) can significantly reduce grain yield of more than 20% [12,13]. All the physiological processes of wheat plants are sensitive to temperature and can be damaged by heat permanently. Heat stress during anthesis can increase floret abortion [14] and temperatures over 30 °C during floret formation may lead to complete sterility [15]. Heat stress during reproductive stage can lead to pollen sterility, tissue dehydration, lower CO_2_ assimilation, increased photorespiration and reduced time to capture resources due to accelerated growth and senescence, consequently reducing the yield [11,16]. Besides the effects on grain yield, heat stress also has great impact on wheat grain quality. It was reported that heat stress could alter the ratio of gliadin to glutenin, which may lead to weaker dough properties and reduce baking quality [17]. However, there was a great diversity of the dough weakening effect under heat stress in other studies [18,19] because wheat quality mainly depends on genotype (G), environment (E) and their interactive effects (G × E) [20]. Several studies carried out at the CIMMYT showed diverse responses of different cultivars in quality traits under drought and heat stressed conditions. Hernandez-Espinosa et al. [21] reported that grain morphology (grain density and size), protein content and flour yield were strongly affected by the environment, while the traits related to gluten quality (gluten strength and extensibility, and bread loaf volume) were mainly determined by genotype, but the environmental and G × E effects were also important, especially for gluten extensibility. Li et al. [22] tested 15 quality parameters and found similar results that environmental factors have large effect on grain yield, while grain hardness and gluten quality-related traits are mainly controlled by genotype. Moreover, they found that drought and heat stress showed contrasting effects on dough rheological properties, where heat stress decreased dough tenacity (increased extensibility), slightly reduced dough strength and increased bread loaf volume while drought stress is the opposite. These results suggest that wheat quality is determined by many inter-related factors.

Although the impact of rising CO_2_ concentration and elevated temperature on plant growth and development has been well studied, the combined effects of heat stress with eCO_2_ on wheat crop performance remain unclear. Stomatal closure is induced by eCO_2_, which leads to the reduction of transpirational canopy cooling; however, higher temperature results in higher water vapor deficits in the air, causing an increased transpiration rate thereby offsetting the effect of stomatal closure induced by eCO_2_ on crop water relations [23]. On the other hand, reduced stomatal conductance under eCO_2_ will slow transpiration rate thereby alleviating the impact of water stress, which is often concurrent with heat stress [24]. However, plants’ ability to sustain leaf gas exchange is dependent on genotype and stress intensity [25]. More detailed studies are required to assess the balance of these processes and how they acclimate to different environmental factors, because under eCO_2_ growth environment plants’ response to these abiotic stresses can be much more complicated. Therefore, this review summarizes current knowledge regarding the effects of eCO_2_ and heat stress on grain yield and quality in wheat and aims to (i) introduce wheat quality traits that are sensitive to abiotic growth conditions, (ii) discuss the impacts of eCO_2_ and heat stress on wheat grain yield and quality and the underlying mechanisms.

## 2. Wheat Quality and Grain Protein

Wheat is the most important staple food in the world due to its wide adaptation to diverse growth conditions and its unique property of gluten protein fraction in the grain. Wheat has evolved itself from emmer wheat into the cultivated species today by both nature and anthropogenic processes since primitive times (ca. 3000–4000 BC). There are two major wheat species nowadays utilized for food production. The first one is common bread wheat (*Triticum aestevum* L.), a hexaploid wheat that is mainly processed into baking products and the other one is tetraploid durum wheat (*T. turgidum* L. var. durum), which is used to make coarse flour (semolina) for pasta making. Wheat species can be classified by grain hardness, color (red, white and amber) and growing season (spring or winter wheat) and within each class, wheat grain can be evaluated by different grading factors [26]. Wheat grain quality is a combination of many specific parameters. The processing and end-use quality is determined by the multiple phenotypic characteristics of grain, flour, dough and final products [27]. Grain protein content (GPC) is considered as one of the most important components affecting the baking quality [28]. Besides, concentration of mineral nutrients, grain hardness (milling properties), grain size, starch content and minor grain constituents such as lipids and soluble proteins also play important roles in determining end-use properties [10,29].

Baking quality mainly depends on the protein composition and concentrations of wheat grains. Wheat grain proteins are divided into three categories according to their solubility in different solvents: (1) water-soluble non-prolamins, albumins and globulins (ALGL), which play important roles in grain metabolism, development and response to environmental factors [30]; (2) gliadins (GLIA), which are soluble in 70% ethanol at room temperature and (3) glutenins (GLUT), which are soluble in dissolving media [31,32]. Gliadins and glutenins are storage proteins, which decide the baking quality, and they are collectively called gluten proteins. Gliadins are mixtures of single polypeptides and are classified into four subgroups: α-, β-, γ- and ω-GLIA, which can be separated by gel electrophoresis at low pH and by reverse phase high performance liquid chromatography (RP-HPLC) [33], whereas glutenins comprise the subunits that are aggregated together by disulphide bonds which are high (HMW-GS) and low molecular weight glutenin subunits (LMW-GS). Gliadins and glutenins are responsible for different biophysical properties where gliadins decide the dough viscosity while glutenins determine the dough elasticity and strength [32,34].

The concentrations and composition of gluten proteins are dependent on both the genotypic (variety) and the environmental factors (climate, fertilization, soil, etc.) [35], especially the availability of N fertilization [3,36]. Wieser and Seilmeier [31] reported that different N fertilization strongly influenced the quantities of gluten proteins where the effect on gliadins was more pronounced than that on glutenins, whereas albumins and globulins were barely affected. Moreover, the proportions of gluten proteins were changed significantly, where the hydrophilic proteins (ω-GLIA, HMW-GS) were increased by higher N supply and hydrophobic proteins (γ-GLIA, LMW-GS) were decreased. Daniel and Triboi [33] showed similar results that the percentage of proteins and gliadins in the flour increased with the increase of N supply and the proportion of ω-GLIA in total gliadin increased with N while the α- and β-GLIA decreased with N fertilization. In durum wheat, heat and/or drought stress during cultivation affect the grain quality attributes. According to Li et al. [37], drought tends to enhance gluten strength through increased lactic acid retention capacity (LARC) and mixograph peak time (MPT), while heat stress tends to decrease these parameters. Guzmán et al. [38] reported that the concentration of micronutrients (Fe and Zn) and flour yellowness (processing and pasta-making quality) in durum wheat were improved by drought but reduced by heat stress except Zn content, which increased under severe heat stress due to “concentration effect” induced by smaller grain. Moreover, heat stress was found to reduce gluten strength (alveograph energy, W) leading to a weakening effect on grain quality in durum wheat. However, in bread wheat, Hernandez-Espinosa et al. [21] did not detect the absolute weakening dough effect under severe heat stressed condition; both drought and heat stress led to higher gluten strength due to the higher protein content level. Joshi et al. [39] found that Fe and Zn concentrations in wheat grains varied across locations and years and are influenced by higher temperature and soil availability of Zn content in 30–60 cm soil depth. However, the effects of growing conditions are only based on quantitative effects, the influences on the structures, quantities and proportions of flour protein groups and the gluten protein types are largely dependent on the variety [31].

## 3. The Effects of eCO_2_ on Wheat Plants and the Mechanisms Behind

Elevated CO_2_ enhances plant growth (productivity and total biomass) through promoting net CO_2_ assimilation rate (A) and improving water use efficiency (WUE) due to reduced stomatal conductance (g_s_) and transpiration in C_3_ plants (e.g., rice and wheat) and therefore leading to a higher yield [40,41]. Wheat grain yield is mainly derived from photosynthates of leaf, stem and ear during the grain filling stage. The translocation of storage of carbohydrates in stem only contributes 5–10% to final grain weight [42], while flag provides major source of photoassimilate to grain during the period of grain development [43].

According to Wang et al. [4], a meta-analysis of the effects of eCO_2_ on wheat physiology and yield showed that eCO_2_ (450–800 ppm) significantly increased A by 33% and decreased g_s_ by 23%, Rubisco total activity by 26% and Rubisco content by 14%, hence increasing grain yield by 24%. However, the yield increase in free-air CO_2_ enrichment (FACE) experiments was 44% less than those obtained in non-FACE (i.e., enclosure) facilities. While Broberg et al. [41] reported that wheat grain yield increased by 26% under eCO_2_ (605 ppm) on an average, mainly through the increase in grain number. Similar results were found in rice where eCO_2_ (627 ppm), on an average, increased rice yields by 23% through increasing grain mass, panicle and grain number, while FACE experiments showed only a 12% increase in rice yield [44]. In addition, Wang et al. [45] reported that eCO_2_ enhanced rice yield by 20% on average, however, the yield responses to eCO_2_ were smaller under FACE conditions (+16%) compared with other methods including greenhouses (+37%), growth chambers (+24%), and open-top chambers (+20%).

Moreover, the type of cultivars also plays an important role in determining the physiological and yield responses of crops to eCO_2_. Lv et al. [46] reported that eCO_2_ enhanced rice yields by 13.5%, 22.6% and 32.8% for japonica, indica and hybrid cultivars, respectively, in FACE experiments. In wheat, eCO_2_ had significantly different effects on A between the two types of wheat cultivars (i.e., spring and winter wheat) with a higher increase (71%) in spring wheat cultivars and only 23% increase in winter wheat cultivars [47]. In addition to grain crops, eCO_2_ was also reported to increase the yield of vegetables, ornamentals, non-agricultural herbaceous species and woody species by 33% on average with a doubling atmospheric CO_2_ concentration [48]. According to a meta-analysis conducted by Dong et al. [49], eCO_2_ (827 ppm) generally enhanced yield of vegetables by 34% mainly through the increasing number of organs and vegetable mass. However, other environmental factors could modulate the eCO_2_ effects on yield responses including temperature, light, water availability and nutrient supply. For example, the eCO_2_ yield stimulation in wheat plants was stronger in the regions with low agronomic productivity [41] and the enhancement in A of wheat and rice plants was greater under sufficient nutrient compared to that under lower nutrient supply [4,44]. Therefore, optimizing growth environments are required to maximize the eCO_2_-induced yield benefit in agricultural systems, such as additional light (PAR) and high N availability [50,51].

Although eCO_2_ can enhance the productivity and yield of agricultural crops, it on the other hand alters and decreases the plant quality, depressing the concentrations of macronutrients (i.e., carbohydrates, protein, and fat) and micronutrients (i.e., minerals, vitamins and phytonutrients) [7,8,52]. Dong et al. [53] reported that eCO_2_ increased the concentrations of fructose, glucose and total soluble sugar in the edible part of vegetables but decreased the concentrations of protein, N, magnesium (Mg), iron (Fe) and zinc (Zn) by 9.5%, 18.0%, 9.2%, 16.0% and 9.4%, respectively. A meta-analysis conducted by Taub et al. [54] on several major food crops showed that eCO_2_ (540–958 ppm) reduced cereal grain protein concentrations by 10–15% on an average in wheat, barley and rice and reduced tuber protein concentration in potato by 14%. Moreover, for soybean, there was a smaller but statistically significant decrease of protein concentration of 1.4% compared with that grown under ambient CO_2_ concentration (aCO_2_). In rice, the nutritive value of grains was also negatively affected by eCO_2_ under FACE conditions through a decrease in protein by 6% and copper (Cu) content by 20% [55].

It is well known that eCO_2_ has direct effect on C and N metabolism in wheat, resulting in changes in the chemical composition in plants [8,56]. In a FACE experiment, Högy et al. [5] reported that eCO_2_ leads to an overall reduction in grain protein concentration by 7.4% in spring wheat cultivars, particularly the N- and glutamine-rich gliadin fraction, thus lowering the gluten concentration and reducing baking quality. Along with the lowered protein concentration in grain, the composition of amino acids and their concentrations were also modified under eCO_2_, and the size distribution was significantly shifted towards smaller grains [5]. Blandino et al. [57] also suggested a 7% decrease of protein content in four winter wheat cultivars, accompanied by a reduction in dough strength of plants grown under FACE conditions. A three-year field trial in Australia demonstrated that eCO_2_ consistently decreased baking quality and grain protein content in wheat, and protein composition changed towards a greater glutenin/gliadin ratio in all years [58]. Moreover, eCO_2_ could also reduce concentration of minerals in wheat grain. For instance, a significant decrease in concentrations of Zn and Fe in wheat grain has been reported [6]. Similar reduction in grain S [59], Ca, Fe and Zn [60] concentrations was documented for wheat grown under eCO_2_.

**Figure 1:**
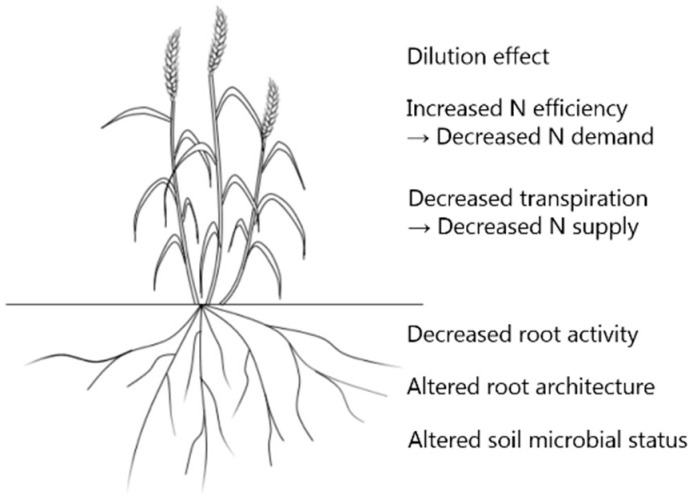
Possible mechanisms explaining the effects of eCO_2_ on wheat grain quality.

There are many different hypotheses explaining these changes of grain quality traits (summarized in Figure 1) and the most frequently mentioned one is the dilution effect, which includes two aspects, biomass dilution and functional dilution [61]. Biomass dilution mainly results from the accumulation of non-structural carbohydrates (NSC) (i.e., soluble sugars, starch, fructans, etc.) which are initial long-term C-storage products of enhanced photosynthesis induced by eCO_2_ and therefore reduces the concentration of other constituents [62]. It means that proteins and minerals are diluted by the increased photosynthetic assimilation of carbon, which poses a great threat to future food security because the calories in the food may be sufficient but undernourishment with essential mineral nutrients. For example, eCO_2_ greatly increased the ratio of C to N by increasing the starch and total NSC concentrations and decreasing grain N concentration in wheat [63]. However, Taub et al. [54] found that the magnitude of the negative eCO_2_ effect on wheat grains was smaller under high soil N conditions than under low soil N with the decrease of grain protein concentrations by 9.8% and 16.4%, respectively, suggesting that the dilution effect may be compensated by a higher nutrient supply. Besides, a relative increase in the synthesis of C-based secondary compounds that are low in N (e.g., lignins, tannins or other polyphenolics) may lead to dilution as well [64], but there was no consistent response [65,66,67].

Functional dilution refers to a decrease in dry mass concentration of N (Nm) due to increased shoot specific activity, which means that N concentration declines due to the accumulation of additional photosynthates by shoots [61]. This theory bases on the functional-balance model:

(1) $$\mathrm{Nm}\;\propto \;\frac{\mathrm{root}\;\mathrm{mass}\;\times \;\mathrm{rate}\;\left(\mathrm{absorption}\right)}{\mathrm{leaf}\;\mathrm{mass}\;\times \;\mathrm{rate}\;\left(\mathrm{photosynthesis}\right)},$$

where the tissue N concentrations are dependent on the relative activities of roots and shoots and the partitioning of photosynthates is controlled by the relative rates of root absorption of soil nutrients and leaf photosynthesis [68,69]. Functional dilution seems to be pervasive because increased photosynthetic rates for plants grown under eCO_2_ is frequently observed if we assume the shoot specific activity equal to photosynthetic rate [70].

Within either biomass dilution or functional dilution, all other mineral elements except C, H and O that are assimilated through photosynthesis should be diluted to a similar ratio. However, there are many heterogeneous responses of different mineral concentrations for crops under eCO_2_. For instance, the decrease in Zn concentrations in rice grains under eCO_2_ was significantly different from those in Cu, Ca, B and P [6] indicating that dilution is not the only mechanism responsible for decreasing nutrient concentrations in plants under eCO_2_ [54,61,71]. Li et al. [72] reported that both the concentrations of K, Ca and Mg in wheat organs and the total accumulations of these elements in the plants were significantly decreased by eCO_2_. Moreover, they also found decreases of the concentration of these minerals in the xylem sap, suggesting that the reduced mineral concentrations were not only because of dilution effect but also due to a reduced nutrient acquisition by roots. Within the functional balance concept mentioned above, decreased specific root activity could also play a role in the reduction of nutrient concentrations in plants through a decreased root uptake rate under eCO_2_, but the effect is larger for plants grown in soil than in hydroponics [73]. It is reported that the average mean decrease of specific root uptake rate of N (i.e., uptake per unit root mass or length) for plants grown in solid media is 16.4% [74,75,76]. While the results for root uptake kinetics in solution were quite different and variable [69], but the overall trend was to increase rather than decrease the specific N uptake [77,78]. Therefore, there are some elements influencing the root uptake that are not present in hydroponics. This indicates that the soil microbiomes, rhizosphere conditions and/or root architecture may play a role in nutrient uptake as well.

When it comes to the acquisition of nutrients, there are two aspects: uptake and demand by the plants. On the one hand, eCO_2_ could affect the ability of soil-root system to supply N, which refers to source effect. On the other hand, eCO_2_ could also increase source use efficiencies, which allows plants to sustain growth in a lower N concentration leading to a lower demand of nutrients (demand effect) [61]. Besides the root specific activity mentioned above, it is widely recognized that the eCO_2_-induced decrease in plant mineral uptake associated with the reduced mass flow or diffusion of mineral ions from the soil solution to the root surface due to lower transpiration rate which results from a reduced stomatal conductance under eCO_2_. There is evidence that eCO_2_ enhances the root growth, which may enable the plants to acquire more nutrients; while a number of studies have also indicated that eCO_2_ depresses root hydraulic conductance [79] probably via down-regulating genes encoding aquaporin hereby reducing the mass flow. In addition, it has been proposed that the effect of transpiration rate on mineral uptake is more pronounced with those primarily transported via mass flow (e.g., N, Ca and Mg) than those via diffusion (e.g., P, K and most micronutrients) [80]. This may lead to a shift in the stoichiometry, further affecting nutritive value of the grain [7]. However, to date these possible effects of eCO_2_ on root water and mineral uptake have not been fully illustrated.

## 4. The Effects of Heat Stress on Wheat Grain Quality

Wheat is a temperate crop adapted to temperatures below 30 °C and the threshold temperature during post-anthesis stage is 26 °C [18]. Heat stress (over 35 °C) has a huge impact on both grain yield and grain quality during anthesis and grain-filling phase; however, most of the studies focused on the heat stress effects on grain yield components [18,81,82], only a few studies investigated the impact of heat stress on grain quality traits [17,18,35,83,84]. Increased temperature affects grain development due to the limitation of assimilate supply, grain-filling duration and rate, and starch biosynthesis and deposition [11]. The effects of heat stress on wheat grain quality are summarized in Table 1. The response of wheat to heat stress varied between genotypes and the degree of heat-caused damage depends on the intensity, duration and frequency of heat stress [83,85].

Although extremely high temperatures have more detrimental impact on grain quality, within moderate or chronic temperature range (15–35 °C), there are also marked changes in grain quality. For example, dough strength (measured by resistance to extension of a dough piece in the Babender Extensograph) was increased with rise in daily average temperature up to about 30 °C; however, when the temperature was above this threshold value to max. 36 °C, even applied for only 3 days, it tended to decrease dough strength. This occurred independent of the timing of the stress, but the degree of reduction varied with different growth stages [89]. In addition, the protein content per grain increases without a change for starch when the temperature rises between 15 and 21 °C during grain-filling stage; however, when temperature reaches 30 °C, the deposition of both protein and starch reduces but with more pronounced decrease in starch than protein [86]. These discrepancies indicate that different growth stages have different threshold and sensitivity to high temperatures. However, the overall higher temperatures generally reduce wheat grain yield through producing smaller grains, reducing grain number and lowering kernel weight [15,90,91], and alter grain quality by increasing GPC but decreasing starch deposition and functional properties of wheat flour [18,35,87], although different wheat genotypes respond differently under heat stress [84,92,93]. For example, Castro et al. [84] evaluated 14 spring wheat genotypes to characterize their response to high temperatures and detected a significant genotype × treatment interaction, suggesting that varieties possess a thermos tolerant response could be used as genetic sources for breeding heat tolerance wheat cultivars.

GPC is the most important characteristic determining wheat grain quality and is in essence determined by the relative rates and durations of protein and starch synthesis [88]. Starch accounting for 65–75% of wheat grain dry weight and over 80% of endosperm weight, which is a decisive factor of grain yield and flour quality [94]. During grain development, starch is deposited into three types of granules differing in size and their formative period in amyloplasts. Large lenticular A-type granules with diameters greater than 15.9 µm are synthesized early during endosperm development; spherical B-type granules with diameters between 5.3 µm and 15.9 µm are produced during mid-development and smaller C-type granules with diameters less than 5.3 µm are initiated late in development [95]. It is reported that high temperatures applied post anthesis reduced the duration of starch accumulation and starch content and modified the size distribution of starch granules with less B-type granules produced in the grain under higher temperatures [88,96]. For the functional properties, starch is composed of two classes of glucose polymers: straight-chained amylose, which is an almost linear α-1,4 glucan molecule comprising 25–30% of grain starch, and highly branched amylopectin, which constitutes 70–75% grain starch [97,98]. Under heat stress during grain-filling stage, the amylose to amylopectin ratio increases, leading to a reduction in dough elasticity [99]. Extension of α-1,4 glucan chains is catalyzed by starch synthases, which are sensitive to heat stress [100], indicating that high temperatures decrease metabolism and enzyme activities involved in starch biosynthesis and reducing the rate of conversion of sucrose to starch [101]. The decreasing starch deposition affects protein concentration by allowing more N per unit of starch [91], leading to smaller grain size, which as a result causing a decrease in milling quality [83].

Under heat stress, the reduction of dough properties of wheat is mainly associated with the reduction of glutenins in gluten proteins especially the HMW-GS [102] that accounts for the genotypic variance in wheat quality [35]. Although there is a general increase in grain protein proportion relative to starch content under high temperatures, due to less temperature sensitivity of N accumulation than starch deposition [88], the protein composition alters towards a poorer flour quality by the following reasons summarized by Blumenthal et al. [102]. First, heat stress decreases synthesis of glutenins therefore leading to the reduction in glutenin/gliadin ratio with gliadin synthesis being maintained or increased [103]. This is explained by the molecular mechanism that there are heat-shock elements (HSE) in the upstream of coding regions of gliadin but not for glutenin [104,105]. However, the effect also depends on genotypes. Stone and Nicolas [18] reported variety difference in glutenin/gliadin ratio in response to heat stress where only one variety (Oxley) showed a decrease in this ratio and Sun 9E-16 increased in this component while the other three varieties had no significant response to high temperature. This suggested that wheat varieties vary in their response of gliadin synthesis to heat stress. The second hypothesis is that heat shocks lower the degree of polymerization of glutenin subunits and reduce the large sized glutenin polymers by altering the formation of disulphide bonds between glutenin peptides therefore weakening the dough properties [106]. In addition, heat-shock proteins (HSP) play an important role in determining the dough-protein function. On one hand, HSP per se is involved in guiding the formation, folding and polymerization of peptides in the grain and can disaggregate and hydrolyze deformed proteins under stress conditions. Therefore, under heat stress, HSP may break off the glutenin synthesis and polymerization thus influencing dough structure [99]. On the other hand, the synthesis of HSP and their prevailing presence in the mature grain could result in the heat-related loss of dough quality. However, according to Blumenthal et al. [102], although the concentration of HSP 70 in mature grain increased under a few days’ heat treatment, there was no strong correlation between the amount of HSP 70 and the loss of dough strength. Moreover, they failed to find the presence of HSE in the upstream coding region of the genes for HMW-GS, suggesting that the weakening of dough properties under heat stress may be more relevant to the degree of polymerization of glutenin molecules and the roles of HSP family during grain development.

Besides GPC, heat stress also affects mineral nutrition of wheat grains with a general decline in the concentration of micronutrients, especially Fe, Zn and Mn [38,107], but the results strongly depend on varieties and meteorological factors. For example, Dias et al. [108] reported that under heat stress, Fe concentration in the stems and leaves decreased in bread wheat but increased in durum wheat, and Mn concentration increased significantly in shoot during grain-filling stage for all genotypes except Golia, which is a less heat tolerant genotype. In addition, Kumar et al. [107] reported that under heat stress, the accumulation of Fe and Mn in flag leaf and spike diminished significantly during booting and grain-filling stages, while some of the varieties showed increased accumulation of micronutrients in either flag leaf or spike. Narendra et al. [109] found a high Zn or Fe content in some heat-tolerant varieties under heat stress, suggesting that these genotypes can be used for future breeding to cope with the problem of malnutrition. Furthermore, it is reported that the proportions of gliadins and polymeric protein were less affected by heat stress in the grain with high Zn concentrations, indicating that grain Zn nutrition may interact with grain-filling temperatures and alter protein composition under heat stress [110].

## 5. The Interactive Effects of eCO_2_ and Heat Stress on Wheat Plants

So far, there are many studies of plant response to single eCO_2_ or high temperature stress, but the research on the combined effect of these two factors on wheat plants is quite limited, especially on grain quality traits. Kadam et al. [111] summarized that the eCO_2_ × high temperature interaction strongly depends on the growth and developmental stages of plants. During vegetative stage, heat stress decreases net CO_2_ assimilation rates in wheat grown under aCO_2_, but eCO_2_ moderates the negative effect of heat stress on canopy photosynthesis. This is because increased temperature will decrease the ratio of solubility of CO_2_ and of O_2_ in water and reduce the specificity of Rubisco for CO_2_ relative to O_2_ [112], leading to a preference to oxygenation rather than carboxylation in C_3_ photosynthesis. Rising CO_2_ concentration can inhibit photorespiration and increase ribulose bisphosphate (RuBp) regeneration capacity thus increasing the net photosynthesis. However, the response varies between species. For example, in rice, leaf photosynthesis increased up to 63%, while in sorghum, the increase was not significant [111]. In wheat, Abdelhakim et al. [113] tested the physiological responses of different spring wheat genotypes of heat-tolerance to eCO_2_ × heat interaction and found that under eCO_2_, all genotypes showed higher photosynthetic rate and maintained maximum quantum efficiency of PSII under heat stress compared to aCO_2_.

Despite the advantage brought by eCO_2_ on the vegetative tissue, during the reproductive phase (especially anthesis and grain-filling stage), temperature is the main factor determining the wheat grain yield and quality when exposed to both eCO_2_ and heat stress [111]. This is due to the irreversible damage of high temperatures to the anabolic and metabolic processes in wheat flowers and grains, which has been mentioned in other studies only focusing on the heat stress effects [11,25]. Chavan et al. [81] reported that although eCO_2_ alleviates the negative impact of heat stress on photosynthesis in wheat, grain yield was reduced equally under aCO_2_ and eCO_2_ by heat stress due to grain abortion and shortened grain filling duration. Moreover, Macabuhay et al. [114] found an increase in stem water-soluble carbohydrates (WSC) under eCO_2_ in both control and heat stressed wheat plants; however, there is limitation of WSC translocation to grains under heat stress, which decreases WSC remobilization. Therefore, the overriding impact of heat stress on crop production seems to limit the potential benefits provided by carbon fertilization of eCO_2_. However, there are some positive responses of wheat in the combined situation (reviewed by Kadam et al. [111]) such as increase in grain yield, number of ears and harvest index. While for thousand grain weight, number of spikelets and grain number, the results were inconsistent, indicating a partial ameliorative effect of eCO_2_ on grain yield when combined with heat stress. Furthermore, varietal difference, seasonal conditions and different experimental treatment applied also contribute to the inconsistency of the plant response under such situation. Besides, the interaction of weeds such as little seed canary grass (*Phalaris minor*) and common lambsquarters (*Chenopodium murale*) in wheat field could accelerate the yield loss under eCO_2_ and thermal scenario due to the similar photosynthetic pathway and nutritional level in weeds and main crop, and the greater response of weeds than crop to eCO_2_ condition [115].

Although eCO_2_ cannot buffer the negative impact of heat stress on reproductive stage, the reduction of grain N concentration under eCO_2_ could be slightly alleviated in heat-treated plants because N accumulation is less sensitive to temperature than C in wheat grain resulting in less carbohydrates relative to N [114]. However, overall, the grain quality seems to decrease in the case of eCO_2_ and high temperature combined situation mainly due to the reduction of storage protein under both conditions [33,116], especially the synthesis of GLUT polymers leading to a diminished dough functionality and baking quality. The effect of eCO_2_ and heat stress interactions on mineral nutrient composition of wheat grains was rarely investigated. However, a study in rice under field conditions showed a significant decrease in grain mineral content, including Ca, Mg, Cu, Fe, Mn and Zn under combined eCO_2_ and heat stress situation across different cultivars. Moreover, some of the micronutrients (Cu, Fe and Zn) were reduced more prominently as compared to eCO_2_ alone [117], suggesting that heat stress may further exacerbate the negative effect of eCO_2_ on grain mineral nutrition.

## 6. Conclusions

Although eCO_2_ increases wheat grain yield, its negative impact on grain quality poses a great threat to human nutrition. This is mainly due to the dilution effect by higher accumulation of photosynthetic assimilates of C and the decreased root N uptake associated with lower transpiration rate and mass flow under eCO_2_. Heat stress generally counteracts the positive effect of eCO_2_ on yield components and may aggravate the negative effect of eCO_2_ on grain quality because wheat is quite sensitive to high temperature stress especially during anthesis and grain-filling stage, which leads to permanent and irreversible damage during flower and grain development. However, grain quality is strongly dependent on variety and environment, and different quality attributes show diverse responses to abiotic stresses. Since eCO_2_ cannot protect wheat plants from high temperature stress, other environmental factors should be taken into consideration, such as enhancing nutrient fertilizing and improving soil water content, etc. Selecting and breeding new genotypes of heat tolerance could be another way to deal with climate change and the increasing demand for food. Moreover, the contemporary crop models for evaluating the effects of different environmental conditions on wheat quality can provide new insights into adaptation strategies to cope with the impacts of climate change on global crop production and grain quality.

## Figures and Tables

**Table 1:** The effects of heat stress on different grain quality traits.

Traits	Impacts	References
Gliadin/glutenin ratio	+/−/ns	[17,18]
Grain protein content (GPC)	+	[21,35]
Starch content	−	[86,87,88]
Dough strength (W)	+/−/ns	[21,37,38]
Gluten extensibility (L)	+	[19,22]
Dough tenacity (P)	−	[22]
Bread loaf volume (LV)	+	[22,38]
Micronutrient concentration (Zn, Fe)	+/−	[38,39]

The “+” sign indicates an increase and the “−” sign indicates a decrease in the performance of the trait of interest.

## Data Availability

The article does not contain original data.
